# Development and Implementation of a Person-Centered, Technology-Enhanced Care Model For Managing Chronic Conditions: Cohort Study

**DOI:** 10.2196/11082

**Published:** 2019-03-20

**Authors:** Curtis L Petersen, William B Weeks, Olof Norin, James N Weinstein

**Affiliations:** 1 The Dartmouth Institute for Health Policy and Clinical Practice Dartmouth College Lebanon, NH United States; 2 Quantitative Biomedical Science Program Geisel School of Medicine Dartmouth Lebanon, NH United States; 3 Department of Epidemiology Geisel School of Medicine Dartmouth College Lebanon, NH United States; 4 Microsoft Healthcare Redmond, WA United States; 5 Medical Management Center Karolinska Institutet Stockholm Sweden; 6 Dartmouth-Hitchcock Medical Center Lebanon, NH United States; 7 Amos Tuck School of Business Dartmouth College Hanover, NH United States; 8 Kellogg School of Management Northwestern University Evanston, IL United States

**Keywords:** mHealth, mobile health, telemedicine, digital biomarker, person-centered care, chronic condition, chronic disease

## Abstract

**Background:**

Caring for individuals with chronic conditions is labor intensive, requiring ongoing appointments, treatments, and support. The growing number of individuals with chronic conditions makes this support model unsustainably burdensome on health care systems globally. Mobile health technologies are increasingly being used throughout health care to facilitate communication, track disease, and provide educational support to patients. Such technologies show promise, yet they are not being used to their full extent within US health care systems.

**Objective:**

The purpose of this study was to examine the use of staff and costs of a remote monitoring care model in persons with and without a chronic condition.

**Methods:**

At Dartmouth-Hitchcock Health, 2894 employees volunteered to monitor their health, transmit data for analysis, and communicate digitally with a care team. Volunteers received Bluetooth-connected consumer-grade devices that were paired to a mobile phone app that facilitated digital communication with nursing and health behavior change staff. Health data were collected and automatically analyzed, and behavioral support communications were generated based on those analyses. Care support staff were automatically alerted according to purpose-developed algorithms. In a subgroup of participants and matched controls, we used difference-in-difference techniques to examine changes in per capita expenditures.

**Results:**

Participants averaged 41 years of age; 72.70% (2104/2894) were female and 12.99% (376/2894) had at least one chronic condition. On average each month, participants submitted 23 vital sign measurements, engaged in 1.96 conversations, and received 0.25 automated messages. Persons with chronic conditions accounted for 39.74% (8587/21,607) of all staff conversations, with higher per capita conversation rates for all shifts compared to those without chronic conditions (*P*<.001). Additionally, persons with chronic conditions engaged nursing staff more than those without chronic conditions (1.40 and 0.19 per capita conversations, respectively, *P*<.001). When compared to the same period in the prior year, per capita health care expenditures for persons with chronic conditions dropped by 15% (*P*=.06) more than did those for matched controls.

**Conclusions:**

The technology-based chronic condition management care model was frequently used and demonstrated potential for cost savings among participants with chronic conditions. While further studies are necessary, this model appears to be a promising solution to efficiently provide patients with personalized care, when and where they need it.

## Introduction

US health care costs continue to rise, driven in large part by the increasing prevalence of chronic conditions and longevity of those afflicted with them [1]. A chronic condition can be broadly defined as a reduction in health that is not transmittable and generally progresses slowly, lasting for an extended period of time [2,3]. Due to these factors, managing chronic conditions is a large burden [4]. With health care spending approaching 20% of gross domestic product and 20% to 40% of health care resources considered wasteful [5-7], reducing the cost of managing chronic conditions is paramount. Some overuse of health care services is attributable to fee-for-service payment systems that require face-to-face encounters for reimbursement [8,9]. While alternative payment models have been designed to mitigate waste [10] by engaging health care providers and their patients in self-care disease management [10], those models still tend to rely on face-to-face visits and irregular and infrequent measurement to manage chronic conditions.

Among those with chronic conditions, reliance on face-to-face visits may delay interventions to mitigate health deterioration until symptoms are acute, accelerating demand for expensive health care services such as hospitalizations, emergency room visits, and unplanned readmissions [11]. This is particularly true for persons with behavioral health issues, where early interventions can reduce the need for acute care [12]. Seemingly, redesign of chronic care management to engage patients in self-care, monitoring for early signs of deteriorating health, intervening early, and avoiding unnecessary care would create value by improving health outcomes and reducing care costs.

Because behavioral and social factors are implicated in over half of premature deaths [13-15], addressing these factors is critical to improving care value. Behavioral change interventions can successfully address those factors [16]; however, because behavioral change is difficult to induce and maintain [12,17], ongoing respectful patient engagement is essential [18]. Technologically enabled real-time information exchange resulting in just-in-time interventions can increase patient feelings of autonomy [19,20], build competence in self-management of chronic diseases [20], and help patients manage their chronic diseases [19,21]. Technology-based solutions like mobile health (mHealth, or health care that uses mobile phones and other mobile devices) have been shown to impact health-related behaviors [22].

Current technology allows for both active and passive collection of digital biomarkers, their subsequent aggregation and analysis, and immediate feedback of information [20,23-25]. With decreasing costs of devices and cloud computing [26], increasing mobile phone penetration [27], and advancements in deep learning, mHealth apps are increasingly being used [28]. While some of these apps have the potential to relieve the need for face-to-face encounters, they tend to focus on a specific disease (or body organ), typically do not interact with patient health care teams, and do not always enhance patient understanding of their health situation. A single app that is integrated with the patient medical record and supported by backend analytics could reduce the burden of interacting with technology while aggregating holistic in vivo data into actionable information for the patient, care team, and health system.

Implementation of mHealth interventions has been studied in a variety of settings and across many disease states [29]. Most studies have examined the disease-specific impact that mHealth apps (developed by academic groups and not designed for widespread use by consumers) have over a short time period within small sample populations [29,30]. To date, mHealth interventions have neither been integrated within a health system nor have allowed for comparison of users with chronic disease states with healthy patients [30]. Further, studies have not examined how support staff are used—a critical aspect of mHealth implementation as payers and care delivery system leaders need to consider workforce impact before they fund such efforts [30]. To address these gaps in the literature, we conducted a retrospective, observational secondary data analysis of an mHealth app and remote care system designed for broad consumer use, determined how and when staff were used to support the system, and calculated the cost impact of the app on individuals with and without chronic conditions.

In the spring 2016, Dartmouth-Hitchcock Health (DHH), an integrated health care system headquartered in Lebanon, New Hampshire, developed a technology- and sensor-based management and health care model called ImagineCare. The model was piloted with volunteer employees who were enrolled in its self-insurance product. Through secondary data analysis we describe the real-world implementation of an mHealth system and sought to determine ImagineCare’s use by—and health care spending impact on—two groups within DHH’s employee population: persons with and without chronic conditions.

This research had two main objectives: to describe the implementation of an mHealth system designed to help monitor and improve the health of an employed population and to examine differences in health services use and costs when comparing those with and without chronic medical conditions.

## Methods

### Developing and Implementing a Technology-Enhanced Care Program

A multidisciplinary team including nurses, physicians, designers, information technology (IT) developers, hospitality specialists, and researchers developed a new care model following an established methodology of disruptive innovation [31]. Over 3 years, the team explored aspects of managing chronic conditions: current care delivery models from the patient and provider perspective, evidence-based guidelines, and behavioral change methods. Devices and software were assessed and selected based on ease of use, technical integration possibilities, cost effectiveness, and clinical relevance.

**Figure 1:**
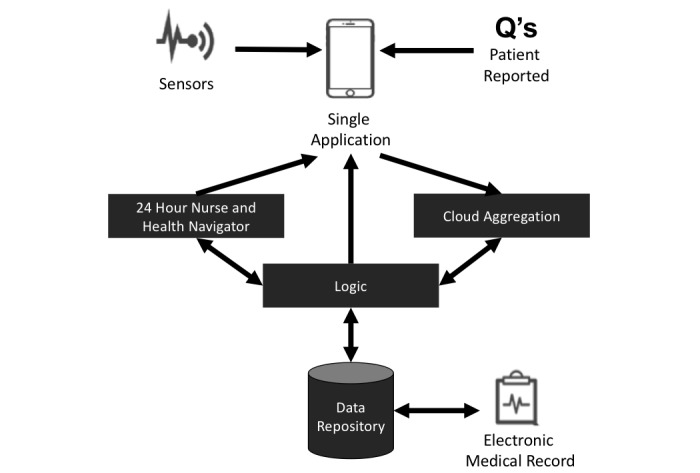
Architecture of ImagineCare care model.

The final ImagineCare delivery model was based on remote monitoring, digital communication, cloud-based analytics, and personal behavioral change support. The model consisted of (1) a 24/7 care support center with staff trained in behavioral change, (2) a clinical workflow application, (3) a mobile app with companion Bluetooth-enabled devices for the participants, and (4) a cloud-based data processing solution (Figure 1).

The care support center consisted of a clinical care team available 24/7 to the participant through text- or voice-based communication. The team was staffed by licensed nurses and health navigators (nonmedical staff trained in customer service and basic health services). Both groups were specifically trained in behavioral change and remote support of patients; they were also coached to provide high-quality customer service designed to keep participants engaged with their health and well-being. Behavioral change training was based on the transtheoretical model of behavioral change, focusing on preparing patients for action and supporting them through plan development and follow-up communication. The staff responded to incoming calls and messages from participants and to alerts that were triggered by collected data. Health navigators passed conversations on to nursing staff based on triage guidelines, clinical judgment, or at the participant’s request. Navigators reached out to participants when cloud-based algorithms identified declining engagement, out-of-normal range monitored vital signs, or negatively trending vital signs.

The iOS-developed app had 3 core functional areas: health data, personal profile, and secure messaging. Health data were collected through sensors, by manual entry, and by participant response to questions. The personal profile section provided valuable context to clinicians by documenting health goals, personal preferences, and social data. The secure messaging function allowed participants to connect to the care team when it suited them best.

To complement personal messages sent by the clinical care team, the system automatically communicated clinical information and suggestions, nudges, and support throughout the app. All system-generated messages used variations on language and collated participant-specific information to make the communication feel personalized. Colors and language were deliberately chosen to support positive behavior change and enjoyment and reduce stress and anxiety. The app itself had either password or biometric security, depending on participant preference.

The system collected data through passive or active encounters with participants, stored data in a cloud-based database, and automatically analyzed them according to medical condition–specific care pathway algorithms. Condition-specific care pathways had been developed through adaptation of systematic reviews of clinical evidence to the patient population via team clinician consensus and by tailoring pathways to enhance self-care opportunities. Safety was held paramount, as no logic made a diagnosis and all decision points were examined by a nurse or health navigator for a final intervention recommendation. All cloud processes and information were stored on Health Insurance Portability and Accountability Act–compliant data services. All algorithms were tested by the developers to ensure patient safety. The app and databases were subject to rounds of vulnerability testing by a third party, ensuring that personal participant information was secure.

Device connectivity, app functionality, and message communication were tested over a 2-month period by 50 healthy individuals. Each device was tested for its ability to connect to the app, collect data, engage algorithms, and respond to both the app and the clinical care team. The testing confirmed that the patient and clinician experiences were good, data collection and algorithm execution were accurate, and no patient safety issues arose.

Voluntary enrollment in ImagineCare began in March 2016, and ImagineCare was launched in May 2016. Volunteers were solicited through emails directed at employees who were insured by DHH’s self-insurance product; 2894 volunteers enrolled, which entailed creating a secure personal account verifying basic information, downloading the mobile app, receiving Bluetooth-enabled sensory devices by mail, and connecting those devices to the mobile app.

The devices that were sent were dependent on the participants’ needed support. For those without a chronic condition, a fitness tracker smartwatch was sent to support general healthy living and wellness, which included collecting data and providing feedback on sleep, physical activity, and mental health. In addition to this support, those with chronic conditions received care and collected data specific to their conditions; for example, blood pressure was measured for those with hypertension, weight was measured for those with congestive heart failure, and blood glucose was measured for those with diabetes.

### Use of the ImagineCare System

To analyze the use of the app for chronic disease management, we classified volunteers into 2 mutually exclusive groups: persons with and persons without chronic conditions. Persons with chronic conditions were defined by documentation of *International Classification of Diseases, Ninth Revision*, codes indicating diabetes, hypertension, congestive heart failure, and/or chronic obstructive pulmonary disease in the prior year. Persons without these codes were defined as without chronic condition. In this pilot project, other chronic conditions were not included, as they were not specifically actionable within the initial implementation of the mHealth system. After comparing demographics for these 2 groups of participants (including a US Census Bureau estimate of the mean family income of each volunteer’s zip code of residence), we evaluated how they used the ImagineCare system in 3 ways. First, we examined their enrollment, over time, and the number of days it took each participant to complete enrollment. Second, we examined how often participants uploaded vital sign data (data collected from any of the peripheral devices that were connected to the app). Third, we evaluated the frequency and timing of conversations that members of each group had with the system. A conversation was defined as a conglomeration of texts and/or phone calls having to do with a particular event; the date and time of the conversation was that of the first interaction regarding the event, whether it was precipitated by participant, staff, or system logic. The purpose of the conversation was recorded by the ImagineCare nurse or health navigator who communicated with the participant at the time of completion of the communication.

### Impact on Care Costs

Finally, for a subset of 1235 volunteers who had been employees for the prior year and remained in the program throughout the entire pilot period, we assessed total allowed health care charges that the participant incurred, partitioned into 3 types of care: hospital, emergency room (ER), and outpatient care (including medications). To make this comparison robust, we used age, sex, hierarchical condition category (HCC) score [32], and chronic condition status to match volunteers to nonparticipating employee controls, using a 3:5 ratio. We conducted a difference-in-difference analysis that compared the intervention period to baseline charges incurred during the same 9-month period in the prior year for participants and controls, for both with and without chronic condition groups.

We used R version 3.4.1 (R Foundation for Statistical Computing) to conduct all analyses. We used Student *t* tests to compare continuous variables and the chi-square test to compare categorical variables; all significance tests used 2-tailed alpha=.05. As charge data were highly skewed and kurtotic, we log-transformed them to conduct statistical analyses; although for ease of interpretation, we also report nontransformed results. This secondary data analysis was approved by Dartmouth College’s Committee for the Protection of Human Subjects (CPHS #30385).

## Results

### Use of the ImagineCare System

Of the 2894 participants in the pilot project, 2518 were considered without chronic condition and 376 were considered to have a chronic condition; participants without a chronic condition were younger and more likely to be female than persons with chronic conditions (Table 1). The 2 cohorts took a similar amount of time to enroll in the program after having been emailed an invitation to do so and had similar estimated annual incomes.

**Figure 2:**
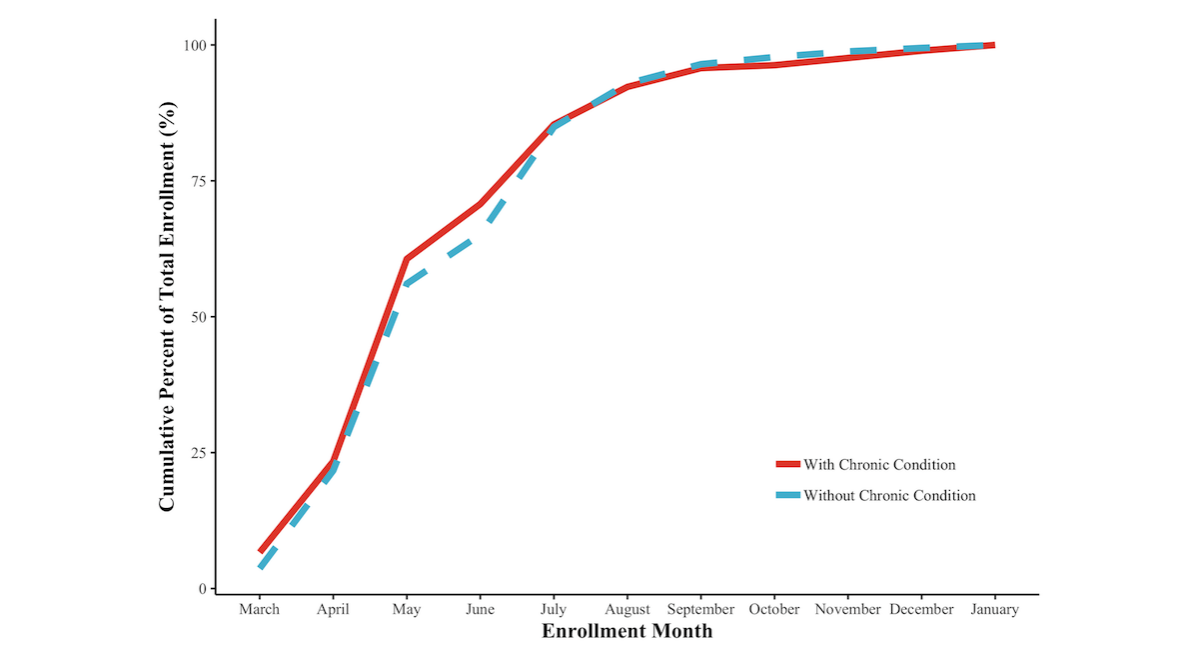
Cumulative enrollment in the pilot program for without chronic condition (dotted line) and chronic condition participants (solid line), March 2016 to January 2017.

**Figure 3:**
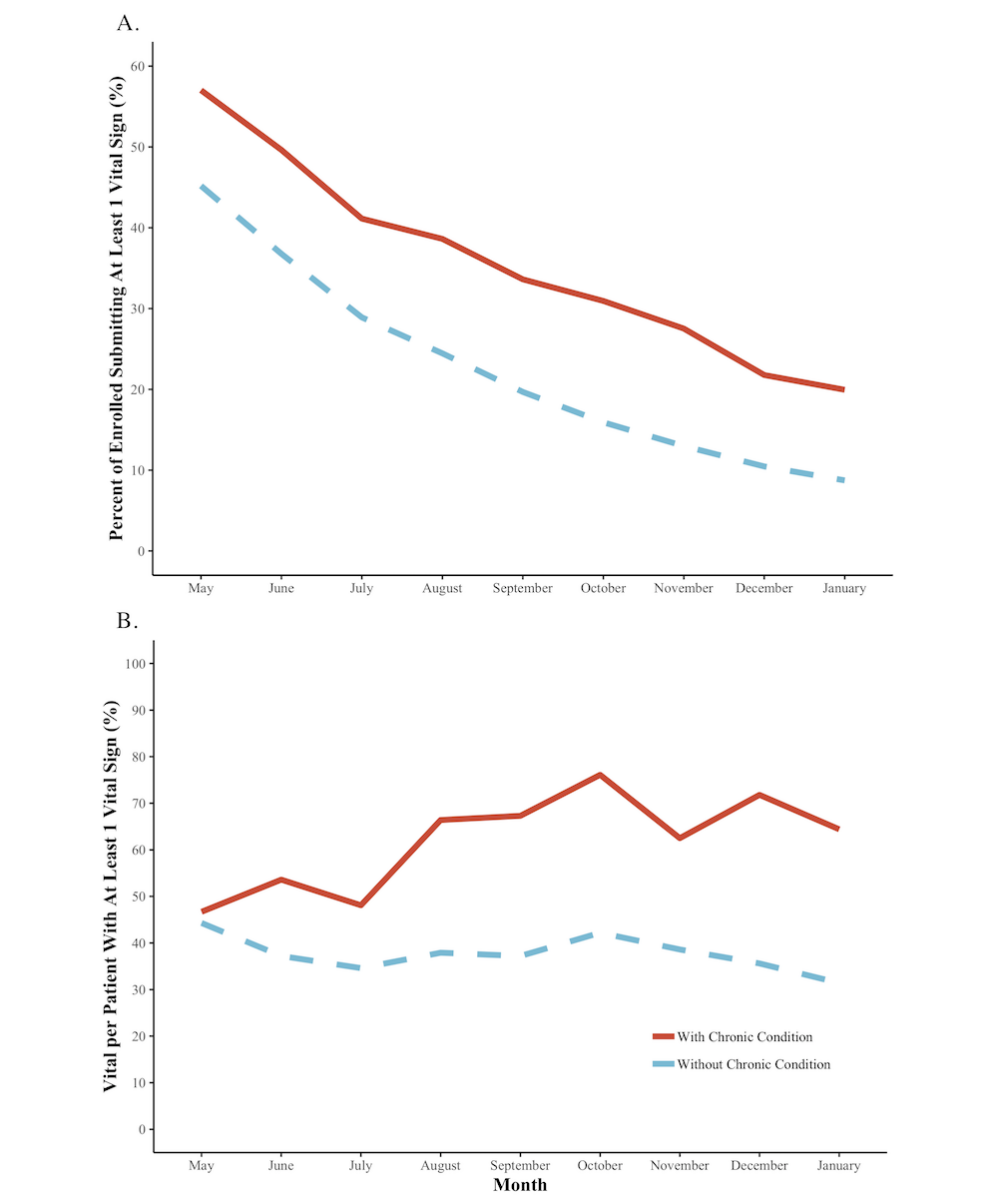
Submissions by without chronic condition (dotted lines) and chronic condition (solid lines) cohorts for each month of the pilot. A) Percentage of enrollees submitting at least 1 vital sign. B) Average number of vital signs submitted (for individuals who submitted at least 1 vital sign that month).

Cumulative enrollment of participants with and without a chronic condition was similar over time (Figure 2). Enrollment was fastest between March and May and slowed somewhat between May and September, when it essentially stopped. The proportion of participants who submitted at least 1 vital sign each month consistently fell for both persons with and without a chronic condition during the pilot, dropping to less than 10% (220/2518, 8.73%) of persons without a chronic condition by the end of the pilot period; persons with chronic conditions remained somewhat more engaged with the system throughout the pilot period, although that engagement waned (Figure 3A). The per capita number of vital signs submitted by engaged patients remained stable for the cohort without a chronic condition but increased somewhat among persons with chronic conditions between May and October before stabilizing (Figure 3B). Although they comprised only 12.99% (376/2894) of the population, persons with chronic conditions contributed 28.34% (62,011/218,794) of all submitted vital signs.

**Table 1:** Characteristics of participants with and without a chronic condition in the ImagineCare pilot.

Characteristics		Chronic condition (n=376)	Without chronic condition (n=2518)	*P* value
Age in years, mean (SD)		52.0 (11.7)	39.3 (12.2)	<.001
**Sex, n (%)**				<.001
	Female	215 (57.2)	1889 (75.0)	N/A^a^
	Male	235 (47.2)	625 (24.8)	N/A
	Other/unknown	6 (1.6)	4 (0.2)	N/A
Days to complete enrollment, mean (SD)		12.0 (33.3)	10.4 (23.6)	.24
Estimated zip code level income ($), mean (SD)		67,500 (21,400)	66,200 (19,700)	.25

^a^N/A: not applicable.

**Table 2:** Number of total conversations and conversations per patient between staff and participants with and without chronic conditions by shift, purpose, and staff role.

Characteristics		Conversations between staff and participants				*P* value
		Chronic condition		Without chronic condition		
		Total, n	Per capita	Total, n	Per capita	
Overall		8587	1.14	13,020	0.32	
**Shift**						
	16:00-24:00	4229	1.73	6001	0.48	<.001
	08:00-16:00	2993	1.16	5159	0.34	<.001
	00:00-08:00	1365	0.53	1860	0.15	<.001
**Purpose**						
	Administrative	3469	6.97	9701	3.85	<.001
	Clinical alert	3198	6.42	263	0.1	<.001
	Health coaching	362	0.73	299	0.12	.012
	Lack of engagement	341	0.68	1190	0.47	.003
	Technical question	938	1.88	1263	0.50	.002
	Other/undetermined	279	0.56	304	0.12	.004
**Team member type**						
	Health navigator	4968	22.36	11,492	0.78	<.001
	Nurse	3619	15.35	1528	0.19	<.001

Persons with chronic conditions also accounted for 39.74% (8587/21,607) of all staff conversations, with higher per capita conversation rates for all shifts (*P*<.001 for all, Table 2). For both with and without chronic condition cohorts, participants engaged in conversations significantly more frequently in later hours, toward the 16:00-24:00 shift (Table 2). Administrative conversations accounted for 74.51% (9701/13,020) of conversations between persons without chronic conditions and with staff; administrative conversations and clinical alerts each accounted for 40.40% (3469/8587) of conversations that persons with chronic conditions had with staff. Conversations were most common with health navigators (16,460/21,607, 76.18%); however, persons with chronic conditions were much more commonly referred to nursing staff (3619/8587, 42.15% vs 1528/13,020, 11.74% for without chronic condition participants, *P*<.001). A total of 70.31% (3619/5147) of all nursing staff conversations were with the chronic condition group as compared to 29.69% (1528/5147) of health navigator staff conversations.

**Figure 4:**
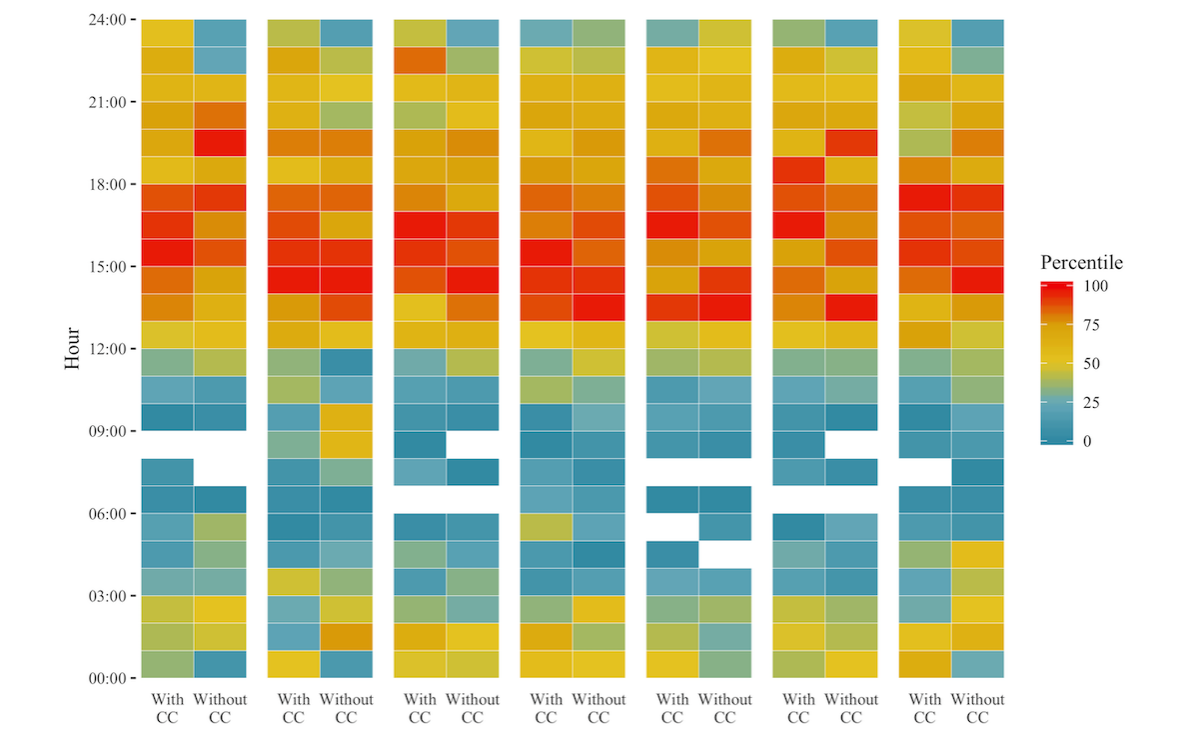
Time of communications by day, hour, and cohort for March 2016 to January 2017. Each week is separated by hours scored by percentile of conversations for that hour. The percentile of conversations (0 corresponding with 0 conversations for that hour) for persons with chronic conditions (With CC) and without chronic conditions (Without CC) are plotted for each hour in each day. The largest concentration of conversations for participants without chronic condition was on Mondays between 14:00-15:00, while it was on Sundays between 15:00-16:00 for persons with chronic conditions.

**Figure 5:**
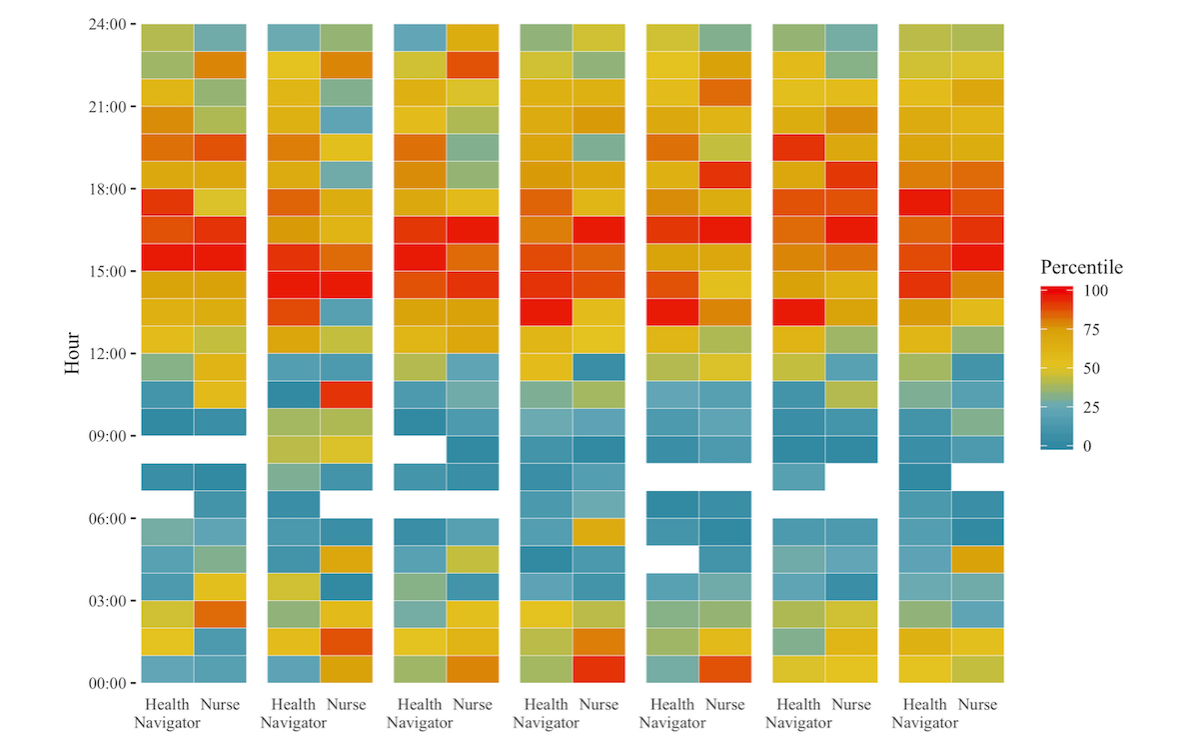
Time of communications by day, shift, and staff role for March 2016 to January 2017. Each week is separated by hours scored by percentile of conversations for that hour. The percentile of conversations (0 corresponding with 0 conversations for that hour) for health navigators and nurses are plotted for each hour in each day. The largest concentration of conversations with health navigators was Mondays between 14:00-15:00; with nurses, it was Sundays between 15:00-16:00.

Patterns of communication across time of day and day of week differed somewhat when comparing with and without chronic condition cohorts. Both groups had their highest concentration of conversations after noon, regardless of day (Figure 4); however, when compared to the without chronic condition group, persons with chronic conditions had relatively more conversations in the late night, and Mondays appeared to be the day on which participants without a chronic condition most frequently communicated with the system. Relatively high concentrations of communications with nurses were more sporadic than those with health navigators throughout the week; however, as the week progressed, health navigators and nurses had similar concentrations of conversations (Figure 5).

There were no reports of adverse health events. System bugs were limited to incorrect responses to question scores, which were investigated and fixed.

### Impact on Care Costs

Our subanalysis that used matched controls to conduct a difference-in-difference analysis of the cost-impact of the new care model found that the variables used for matching were similar for participants and controls for with and without chronic condition cohorts with the exception of prior period ER charges, which were higher for participants without a chronic condition than for matched controls (*P*=.01; Table 3). Our difference-in-difference analysis found that program participation was associated with trends toward lowered ER charges for the without a chronic condition cohort (29% reduction, *P*=.08) and lowered outpatient (20% reduction, *P*=.052) and total charges (16% reduction, *P*=.06) for the cohort with chronic conditions (Table 4).

**Table 3:** Comparison of the characteristics and pre- and postintervention charges for program participants and matched controls in the with and without chronic condition cohorts (actual mean values are provided, but *P* values for charge data are based on results using log-transformed data).

Characteristics		Chronic condition			Without chronic condition		
		Program participants (n=193)	Matched controls (n=343)	*P* value	Program participants (n=1042)	Matched controls (n=1730)	*P* value
Age in years, mean		51.1	51.9	.37	39.7	40.3	.17
Male, n (%)		70 (36.3)	125 (36.4)	.97	274 (26.3)	446 (25.8)	.77
HCC^a^ score		4.13	4.6	.16	1.77	1.69	.30
Prior period, mean							
	Acute care charges ($)	1545	1674	.74	274	771	.45
	ER care charges ($)	296	356	.17	191	156	.01
	Outpatient charges ($)	8456	7533	.07	3970	4773	.82
	Total charges ($)	10,297	9563	.09	4435	5700	.74
Intervention period, mean							
	Acute care charges ($)	1305	1178	.73	317	548	.89
	ER care charges ($)	252	479	.06	192	213	.89
	Outpatient charges ($)	8402	9159	.59	5201	5192	.70
	Total charges ($)	9959	10,816	.53	5710	5953	.65

^a^HCC: hierarchical condition category.

**Table 4:** Results of the difference-in-difference charge analysis for with and without chronic condition cohorts showing only the difference-in-difference statistic. The regression included the following variables: age, sex, HCC score, time, case, and time*case (which represents the difference-in-difference statistic after adjusting for the other variables). Where *P*<.10 for the natural log of the charge categories, 95% confidence intervals (CIs), and the *P* value and coefficient (β) for non–log-transformed charges are provided; if *P*≥.10, cells are left blank. Neither the with nor the without chronic condition cohort had difference-in-difference statistics that were *P*<.10 for acute care.

Type of charge	Chronic condition				Without chronic condition			
	β LN^a^ charge	95% CI	*P* value	β^b^ charge $	β LN charge	95% CI	*P* value	β charge $
Acute care	—	—	—	—	—	—	—	—
ER^c^ care	—	—	—	—	–0.2	–0.422 to 0.022	0.08	–56
Outpatient	–0.43	–0.868 to 0.004	0.05	–1679	—	—	—	—
Total	–0.44	–0.884 to 0.009	0.06	–1590	—	—	—	—

^a^LN: natural log.

^b^Coefficient for non–log-transformed charges.

^c^ER: emergency room.

## Discussion

### Principal Findings

Within its self-insured employee population, DHH designed, piloted, implemented, and studied a remote monitoring system designed to improve patient self-management of their health status, particularly for persons with chronic conditions. We found that persons with or without chronic conditions signed up for the volunteer program similarly; however, those with chronic conditions were older and more likely to be male. Persons with chronic conditions used ImagineCare differently than did participants without a chronic condition: although both groups used the system less as time progressed, persons with chronic conditions appeared to be actively engaged with the system for a longer time period. Engagement fell considerably in both groups over the pilot period at similar rates. Interestingly, even at the beginning of the pilot, only about half of the participants in either cohort were actively engaged with the system. Finally, among persons with chronic conditions, our difference-in-difference analysis uncovered a substantial potential for reductions in care costs when compared to matched controls, while the participants without chronic conditions did not demonstrate such large charge reductions.

Our results suggest that effort needs to be expended to engage patients in the use of mHealth apps. While the engagement we experienced was higher than that found in a Federally Qualified Health Plan [33], several approaches might improve system engagement going forward. First, evaluation of participant eHealth literacy—the ability of patients to communicate through written text, a working knowledge of computers or mobile phones, and a basic understanding of their health and treatment [34,35]—might have helped to target participants who lacked such understanding or demonstrate to them that use of the system might have been beneficial [36]. While the observed higher engagement might have resulted from the testing process and user-centered design, the engagement might have been improved by ensuring that there was a good fit between the app, end users, recruitment approach, and treatment process [33]. Nonetheless, the relatively low engagement suggests that more should be done to encourage customers of such systems to use technology to monitor and manage their health.

Our results suggest that care models like ImagineCare can be integrated into traditional health care delivery systems but that a focus on enrolling patients with chronic conditions would be wisest: they are more likely to remain engaged and have the greatest potential to generate cost savings. While the pilot effort was successful, there were substantial technical challenges in managing and coordinating data input from a multiplicity of systems, which, as others have noted, highlights the lack of standards for technology interoperability in implementing mHealth programs [37]. Not only were data collection and storage difficult because of the need to integrate firmware, schema, and data types, ongoing data analytics overwhelmed the initial computational power provided to run the system. Much of the system’s success relied on difficult integration of devices that traditionally run on third-party applications; this creates an opportunity for device manufacturers to produce devices with open software development kits. Should health care organizations want to develop similar care models, they should use existing technologies and leverage existing capacity for large data management and analysis.

Appropriate technical and clinical staffing levels are also necessary to effectively and efficiently run such systems; staffing levels must be adapted to meet the needs of particular patient populations by shift, day, and time since enrollment. Given the high variability in the type and time of communications, the timing of communications during a week, and the decline in communications over time, flexible scheduling based on system engagement may be most efficient [33,38,39]. Our results indicate that systems managing a higher proportion of persons with chronic conditions might require more nursing staff, particularly in later shifts and later in the week.

The use of remotely collected data that monitors health and behavior is an emerging area of research [40]. Such data could be considered digital biomarkers [41]—objective information that can be used to predict changes in health status. It is difficult, costly, and time consuming to collect, process, and analyze nondigital predictors [42,43], and the use of digital biomarkers offers a more efficient method of identifying such markers as the use of devices continuously collecting data increases. One critical requirement in the development of digital biomarkers is connecting these novel measurements to health outcomes [41]. In the context of accelerating US health care spending [44] and private endeavors to address spending growth [45], care models that can use digital biomarkers might have market advantages.

The potential cost savings due to a remote monitoring care system could be highly dependent on the payment model of the implementing health system. Fee-for-service models may have the least to gain as the goal of remote monitoring care is to reduce the use of standard face-to-face services through prevention and point of need interventions. Payment models such as accountable care organizations, value-based models, or those that use bundled payment structures might have much greater savings.

### Limitations

Our analysis has several limitations. First, ours was an open study and our cost comparison used retrospectively matched controls. To better evaluate the impact of such systems on the health of the population and care costs, future studies should prospectively identify control groups and concurrently collect data from them. Second, our definition of having a chronic condition was limited. We believe that this potential bias was minimal as HCC scores between the cohorts were meaningfully different, and we observed differences in use. If such a threat to internal validity were large, observed differences would be biased to the null. Third, more in-depth analyses of how patients used the system would be valuable. For instance, analysis of time spent interacting with the system, responses to system-generated automated messages, eHealth literacy, and measures of patient engagement would be valuable. Fourth, we did not evaluate the impact of the system on clinicians within the health care delivery system; analysis of their ability to integrate data obtained from such programs into their clinical decision-making processes and patient encounters would be valuable. Additionally, we only examine the use of the monitoring model and not the health status of participants measured by it. Fifth, we examined an employed population; while some of them had a chronic condition, they remained employed throughout the study. Findings may not generalize to patients with chronic conditions that preclude their ongoing employment. Finally, we analyzed the implementation of a single monitoring model in a single organization for a relatively brief time period; longer studies including nonemployed patients is needed to gather more knowledge about the use of remote monitoring systems in health care.

### Conclusions

Our results suggest that persons with and without chronic conditions used a remote monitoring care model differently and that their needs for support within such systems differed. This new care delivery model showed promising results, but the long-term success will depend on sustainably engaging patients to participate in the system, developing triage structures that meet patient and health system needs, and appropriately staffing the system so patients get the care that they want and need, nothing more and nothing less.

## Abbreviations

DHH: Dartmouth-Hitchcock Health
ER: emergency room
HCC: hierarchical condition category
IT: information technology
mHealth: mobile health
